# Autophagy as a dual-faced host response to viral infections

**DOI:** 10.3389/fcimb.2023.1289170

**Published:** 2023-12-06

**Authors:** Huanjie Zhai, Tao Wang, Di Liu, Li Pan, Yuan Sun, Hua-Ji Qiu

**Affiliations:** State Key Laboratory for Animal Disease Control and Prevention, Harbin Veterinary Research Institute, Chinese Academy of Agricultural Sciences, Harbin, China

**Keywords:** autophagy, viruses, interferons, viral infection, viral replication

## Abstract

Autophagy selectively degrades viral particles or cellular components, either facilitating or inhibiting viral replication. Conversely, most viruses have evolved strategies to escape or exploit autophagy. Moreover, autophagy collaborates with the pattern recognition receptor signaling, influencing the expression of adaptor molecules involved in the innate immune response and regulating the expression of interferons (IFNs). The intricate relationship between autophagy and IFNs plays a critical role in the host cell defense against microbial invasion. Therefore, it is important to summarize the interactions between viral infections, autophagy, and the host defense mechanisms against viruses. This review specifically focuses on the interactions between autophagy and IFN pathways during viral infections, providing a comprehensive summary of the molecular mechanisms utilized or evaded by different viruses.

## 1 Introduction

Autophagy is an evolutionarily conserved process that maintaining intracellular homeostasis by eliminating dysfunctional organelles, protein aggregates, and senescent proteins (Levine and Kroemer, 2008). When pathogens (including DNA viruses, RNA viruses, bacteria, and parasites) infect host cells, they can induce immune responses such as autophagy within host cells (Kimura et al., 2017; Liu et al., 2019; Xiao and Cai, 2020; Wu et al., 2023).

There is an intricate interplay between autophagy and viral infections. Autophagy aids in the clearance of viruses within infected cells, suppresses viral replication, and regulates the host immune response to limit viral spread. However, it is noteworthy that most viruses have evolved strategies to exploit the host autophagy pathway for efficient infection. By utilizing autophagy, these viruses enhance their survival and replication while evading immune responses and drug therapies (Waisner and Kalamvoki, 2019). This dual role of autophagy in viral infections highlights its complexity and underscores the need for a deeper understanding of its mechanisms and regulation. This review is to comprehensively discuss the diverse molecular mechanisms employed by viruses to either exploit or evade the autophagic responses and type I interferons (IFNs) of the host cell, which may facilitate the development of novel therapeutic strategies specifically tailored to combat viral infections.

## 2 Diverse and multifunctional autophagy

### 2.1 The types of autophagy

Autophagy can be categorized into three types based on the mechanism that mediates the delivery of intracellular components to lysosomes: macroautophagy, microautophagy, and chaperon-mediated autophagy (CMA) (Orenstein and Cuervo, 2010; Mizushima et al., 2011). Macroautophagy delivers cytoplasmic cargo to the lysosome via a double membrane-bound autophagic vesicle, undergoes a process of nucleation, amplification, and closure, ultimately leading to its fusion with lysosomes to form autophagic lysosomes (ALs) (Feng et al., 2014). Microautophagy involves the direct engulfment, and degradation of cargo through the fusion of lysosomes and late endosomes (Uttenweiler and Mayer, 2008; Oku and Sakai, 2018; Wang et al., 2023). CMA is a highly selective form of autophagy that relies on chaperone proteins to specifically degrade target proteins with a unique recognition pentapeptide motif (KFERQ-like). The lysosome-associated membrane protein 2A (LAMP-2A) recognizes the exposed KFERQ group of the binding protein and directs the target protein for lysosomal degradation (Kaushik and Cuervo, 2018; Bourdenx et al., 2021).

Autophagy is divided into two categories based on the selectivity of degradation substrates: non-selective autophagy and selective autophagy (Takats et al., 2019; Martens and Behrends, 2020). Non-selective autophagy, induced by rapamycin treatment and other conditions, has no selectivity for the substrate of degradation (Russell et al., 2014; Grumati and Dikic, 2018). Specific autophagy receptors are involved in selective autophagy processes, such as ubiquitin (Ub)-dependent selective autophagy receptors (SARs), including SQSTM1/p62, neighbor of BRCA1 gene 1 (NBR1), TAX1BP1, CALCOCO2/NDP52, OPTN, AMBRA1, CCDC50, and CCPG1 (Kirkin et al., 2009; Matsumoto et al., 2011; Verstrepen et al., 2011; Khaminets et al., 2016; Viret et al., 2018; Hou et al., 2021; Zhou et al., 2021). Ub-independent selective autophagy receptors include BNIP3, PHB2, NIX/BNIP3L, FAM134B, FUNDC1, TBC15, TBC1D5, STBD1, and UIM-type autophagy receptors (Zhang and Ney, 2009; Jiang et al., 2011; Chen et al., 2016a; Borg Distefano et al., 2018; Marshall et al., 2019; Jiang et al., 2020; Yan et al., 2020). Depending on the autophagic cargo, selective autophagy can be further categorized into different types, including proteaphagy (for proteasomes) (Bartel, 2015; Beese et al., 2019), mitophagy (for mitochondria) (Zhang et al., 2022b), ER-phagy (for endoplasmic reticulum, ER) (Mochida and Nakatogawa, 2022), glycophagy (for glycogen) (Zhao et al., 2018), lipophagy (for lipid droplets) (Singh et al., 2009), nucleophagy (for nucleus) (Papandreou and Tavernarakis, 2020), lysophagy (for lysosomes) (Eapen et al., 2021), pexophagy (for peroxisome) (Zheng et al., 2022), ribophagy (for DNA or RNA phagocytosis) (An et al., 2020), aggrephagy (for protein and RNA aggregates) (Pankiv et al., 2007), ferritinophagy (for ferritin) (Mancias et al., 2015), and xenophagy (for pathogens) (Papadopoulos et al., 2020).

Autophagy is a tightly regulated and stress-induced catabolic pathway that consists mainly of typical and non-canonical autophagy pathways. Typical autophagy is more formally known as macroautophagy, and the non-canonical autophagy pathway is a subclass of autophagy. Instead of relying on intact autophagosomal progenitors, non-canonical autophagy utilizes specific autophagosomal proteins for membrane modification. One form of non-canonical autophagy is known as LC3-associated autophagy (LAP), which is particularly reliant on rubicon proteins. The interaction between rubicon and phosphatidylinositol 3-kinases (PI3Ks) triggers the initiation of LAP (Durgan et al., 2021). Furthermore, the Golgi complex is a potential membrane platform for non-canonical autophagy. The V-ATPase plays an important role in this process (Gao et al., 2016).

### 2.2 The process of autophagy

**Figure 1:**
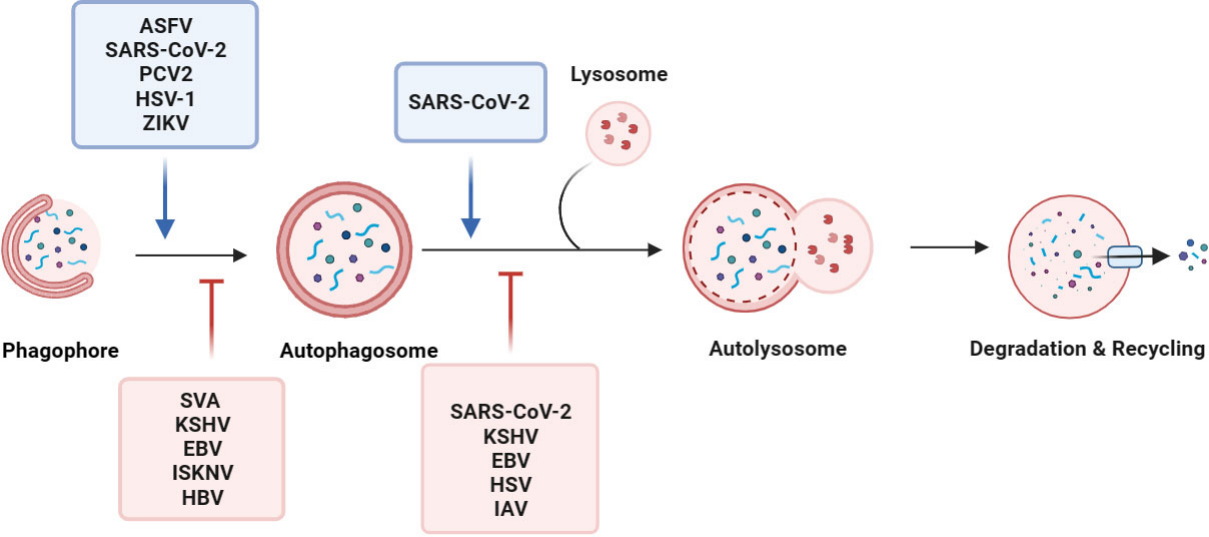
Viruses manipulate the autophagy process. Macroautophagy consists of five sequential stages: initiation, membrane nucleation, phagosome formation and expansion, the lysosomal fusion, and degradation. Viruses, such as ASFV, SARS-CoV-2, and PCV2, regulate the initiation of autophagy, membrane nucleation, phagosome formation, and promote autophagy. For example, the SARS-CoV-2 ORF7a protein triggers autophagy via the AKT-mTOR-ULK1-mediated pathway. STING induces autophagy by inducing the transformation of LC3-I into LC3-II after ZIKV infection of drosophila brain. By inhibiting the activity of the autophagy receptor SQSTM1/p62, EBV and KSHV selectively regulate autophagy. Some viruses evade autophagy by reducing the fusion of autophagosomes and lysosomes in host cells. During SARS-CoV-2 replication, the viral protein ORF7a prevents the fusion of autophagosomes and lysosomes by activating caspase 3 to degrade SNAP29.

Generally, autophagy requires five consecutive steps: initiation, double membrane nucleation and phagosome formation, phagosome elongation and cargo sequestration, fusion of autophagosomes with lysosomes to form autolysosomes, and cargo degradation. The autophagy-related (Atg) proteins function as macromolecular complexes at different steps of the autophagy process, including the ULK complex (also known as the autophagy initiation complex, including ULK1/2, Atg13, FIP200, and Atg101) (Hara et al., 2008; Nakatogawa et al., 2012; Li et al., 2020; Yao et al., 2020), Atg9/Atg9-containing vesicles (Kotani et al., 2018), the class III phosphatidylinositol 3-kinase complex (PI3KC3) (including PI(3)KC3, p150, beclin 1, Atg14L, UVRAG, BIF-1, and rubicon) (Seaman et al., 1997; Hamasaki et al., 2013; Dikic, 2017; Chen et al., 2021), and the Atg12-Atg5 conjugation system (Fan et al., 2011; Fletcher et al., 2018), and the Atg8-PE conjugation system (Geng and Klionsky, 2008; Choy et al., 2012; Tsuboyama et al., 2016). Specifically, the activated ULK1 initiation complex binds Vps34/PIK3C3-Vps30/BECN1 and phosphatidylinositol 3-phosphate (PtdIns3P) to form a complex that favors phagosome extension. The Atg12-Atg5 binds system mediate phagocytic vesicle elongation, sequestering the contents inside the autophagosome and possibly regulating fusion with the phagosome. Rab GTPase and SNARE mediate the fusion of autophagosomes with endosomes, multivesicular bodies (MVB), or lysosomes (Levine et al., 2011; Fletcher et al., 2018). Autophagosomes mature to fuse with vesicles from endosomal systems, including early and late endosomes. Finally, autophagosomes fuse with lysosomes to form autolysosomes, which utilize hydrolytic enzymes in lysosomes to degrade autophagic cargo (Chen et al., 2021) (**Figure 1**).

## 3 Autophagy regulates innate immunity and effects the replication of DNA viruses

### 3.1 Interplay between cGAS-STING and autophagy impacts viral replication

dsDNA serves as a trigger for the innate immune response, leading to the production of type I IFNs and IFN-stimulated genes. Upon DNA binding, cGAS generates cGAMP, which in turn binds to STING (Sun et al., 2013). The cGAMP-STING interaction activates the kinases IKK and TBK1 to induce IFNs production (Wu et al., 2013; Wu and Chen, 2014; Crowl et al., 2017). Indeed, several studies have demonstrated that STING also can activate autophagy. Following viral infection, cGAS can directly trigger autophagy, and the presence of cGAMP or poly(dA:dT) can stimulate STING to induce autophagy (Liu et al., 2019; Zhao et al., 2021). Upon binding to cGAMP, STING is translocated to the endoplasmic reticulum-Golgi intermediate compartment (ERGIC) and the Golgi. The STING-loaded ERGICs serve as a membrane source for LC3 lipidation, a critical step in autophagy. The cGAMP triggered the LC3 lipidation process via WIPI2 and Atg5, which further explains the molecular mechanism by which STING activates autophagy (Gui et al., 2019; Wang et al., 2021b). Selective autophagy is involved in the cGAS-STING signaling pathway. For example, cGAS is significant in recognizing DNA, and the autophagic cargo receptor SQSTM1/p62 can target and degrade K63-linked cGAS, inhibiting the cGAS-STING signaling (Chen et al., 2016b). The K63 ubiquitination of STING leads to the autophagic degradation of STING, preventing the excessive activation of the cGAS-STING signaling and reducing the type I IFN response. Meanwhile, the TBK1 phosphorylates and activates the IFN regulatory factor 3 (IRF3), which leads to the dimerization of IRF3 into the nucleus and the production of type I IFNs (Andrejeva et al., 2004).

#### 3.1.1 The cGAS-STING pathway inhibits viral replication by inducing autophagy

cGAMP-induced autophagy is important for clearing DNA from the cytosol, although it can enhance the replication capabilities of certain viruses (Sun et al., 2013). Studies have found that several proteins of African swine fever virus (ASFV) promote viral replication by inducing autophagy and negatively regulating the production of type I IFNs. For example, the ASFV A137R protein negatively regulates the autophagy-mediated lysosomal degradation of the cGAS-STING-mediated IFN-*β* signaling pathway by targeting TBK1. Further studies revealed that deletion of this protein leads to a significant reduction in the virulence of the ASFV Georgia strain (Gladue et al., 2021; Sun et al., 2022). In addition, the ASFV MGF-505-7R protein interacts with STING, inhibits the cGAS-STING pathway at the level of STING, and degrades STING by promoting the expression of ULK1 (Li et al., 2021). The *MGF-505-7R* deletion in the ASFV genome leads to more IFN-*β* and less viral replication in porcine alveolar macrophages (Li et al., 2021).

**Figure 2:**
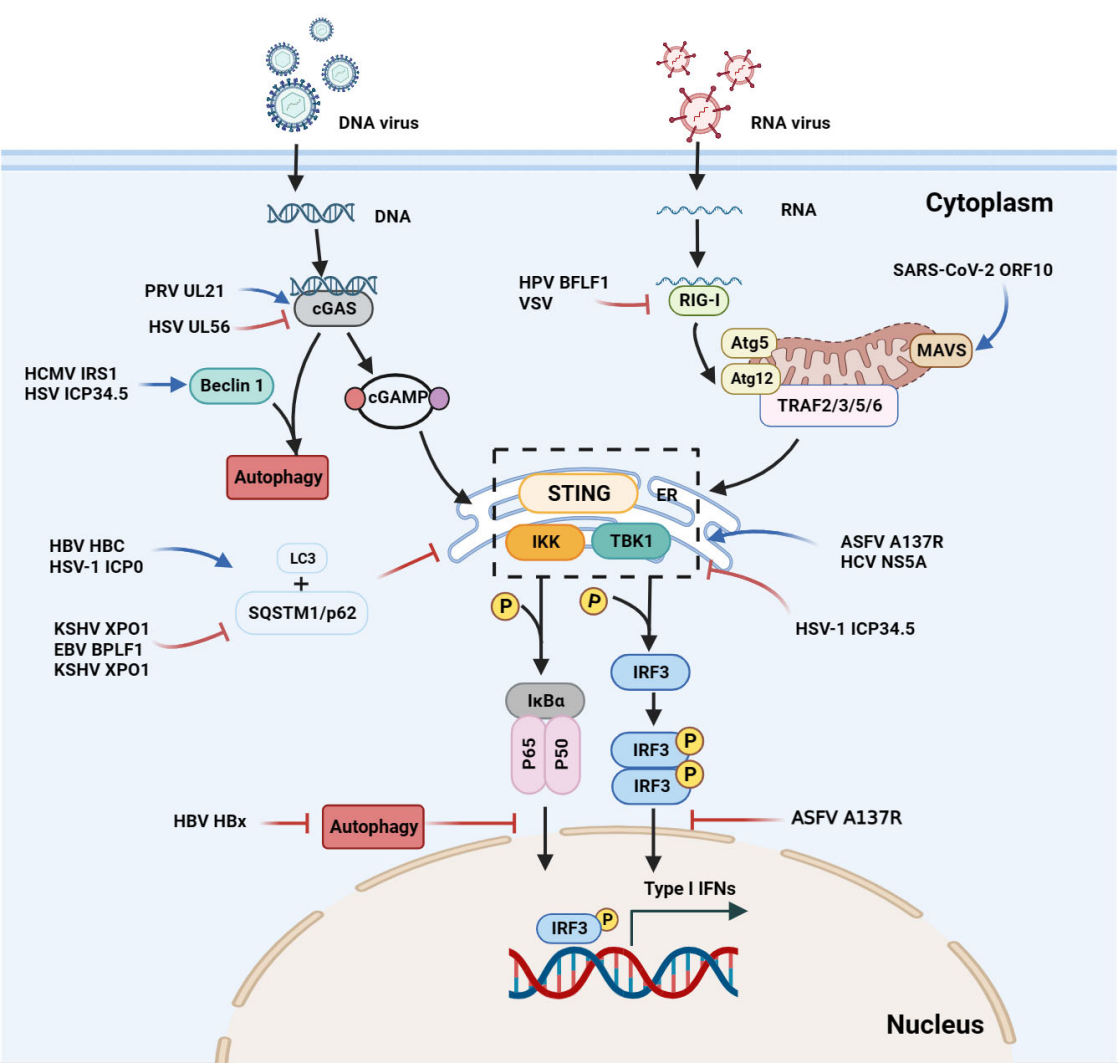
Interactions between autophagy and the interferon signaling pathway during viral infections. Upon DNA binding, cGAS generates cGAMP, which in turn binds to STING. The cGAMP-STING interaction activates the kinases IKK and TBK1, and thus induces IFN production. In the case of ASFV infection, the A137R protein negatively regulates the autophagy-mediated lysosomal degradation of the cGAS-STING-mediated IFN signaling pathway by targeting TBK1. During KSHV infection, XPO1 inhibits SQSTM1/p62 aggregation in the nucleus, thereby enhancing the activation of TBK1 and IRF3 and promoting IFN production. During HSV infection, beclin 1 interacts with the dsDNA sensor cGAS to inhibit IFN-*β* production in the cells stimulated by dsDNA or infected with HSV. Upon HSV-1 infection, the unconventional K27 of the TRIM23 ARF domain is linked to ubiquitination to activate TBK1, promote p62 phosphorylation, and degrade ubiquitin proteins through the autophagy-lysosomal pathway. The ASFV A137R protein negatively regulates the autophagy-mediated lysosomal degradation by targeting TBK1. During HPV-1 infection, the interaction between cGAS and beclin 1 can not only prevent the production of IFNs by inhibiting the synthesis of cGAMP, but also prevent the excessive activation of cGAS by enhancing the autophagic degradation of the virus to maintain the systemic immune balance. SARS-CoV-2, SVA and VSV induce mitophagy-mediated MAVS degradation.

Selective autophagy recognizes and degrades specific cargo labeled with ubiquitination by the E3 ubiquitin ligase family proteins (van Gent et al., 2018), including the tripartite motif (TRIM) proteins, which are key regulators of innate immunity. Some TRIM proteins are involved in the regulation of type I IFNs through cytokine (such as IFN and TNF-*α*) signaling and cytoplasmic PRR (such as MAD5 and RIG-I) (Versteeg et al., 2014). Herpes simplex virus (HSV) replication is enhanced in *TRIM23^−/−^* mouse embryo fibroflasts (MEFs). Specifically, the unconventional K27-linked autoubiquitination of the ARF domain of TRIM23 activates TBK1, promotes SQSTM1/p62 phosphorylation, and degrades ubiquitinated proteins through the autophagy-lysosomal pathway. TRIM23 is essential for autophagy-mediated restriction of a wide range of viruses, an activity that is dependent on its RING E3 ligase and ADP-ribosylation factor (ARF) GTPase activities (Sparrer et al., 2017) (**Figure 2**).

The SQSTM1/p62-mediated autophagy plays a critical role in selectively degrading RIG-I and STING (Xie et al., 2022). Moreover, the Kaposi's sarcoma-associated herpesvirus (KSHV) XPO1 can inhibit induced retention of autophagy adaptor protein SQSTM1/p62 aggregation in the nucleus, thereby enhancing TBK1 and IRF3 activation and type I IFN signaling, leading to inhibit KSHV replication (Meng and Gao, 2021).

#### 3.1.2 Autophagy facilitates viral replication through the cGAS-STING pathway

Autophagy can indirectly attenuate the cGAS-STING signaling pathway, thus preventing excessive activation of cGAS and sustained immune stimulation (Liang et al., 2014). In cells infected with HSV-1, beclin 1 interacts with cGAS, and inhibiting cGAMP synthesis and type I IFN production. Autophagy-mediated degradation removes cytoplasmic pathogen DNA detected by DNA sensors and impairs the transduction of the cGAS-STING signaling pathway (Liang et al., 2014). Furthermore, the HSV-1 UL21 and pseudorabies virus (PRV) proteins induce the E3 ligase UBE3C-mediated K27-linked ubiquitination of cGAS, and the UL21 protein interacts with the selective autophagy receptor TOLLIP to form a complex that recognizes and degrades ubiquitinated cGAS through the autophagy-lysosomal pathway, ultimately inhibiting type I IFNs to promote viral replication (Ma et al., 2022).

TRIM proteins are involved not only in the regulation of type I IFNs, but also in selective autophagy to recognize autophagic cargo (Versteeg et al., 2014; van Gent et al., 2018). The herpesviral BPLF1 forms a three-molecule complex with TRIM25, 14-3-3 and promotes the self-ubiquitination of TRIM25. The 14-3-3 and TRIM25 complexes are involved in the innate immune response by the K63-linked ubiquitination of RIG-I. BPLF1 blocks type I IFN response by impairing the ubiquitination of RIG-I (Gupta et al., 2018; Gupta et al., 2019). During HSV-1 infection, UL56 interacted with cGAS and inhibited its DNA binding and enzymatic activity (Zheng et al., 2022), and the TRIM9 protein promotes innate immune responses and affects HSV-1 replication (Ozato et al., 2008; Qin et al., 2016). TRIM21 can bind SQSTM1/p62, and mediates the autophagic degradation of the IRF3 dimer and inhibits the type I IFN response triggered by the cGAS-STING pathway (Kimura et al., 2015). By inhibiting STING/IRF3 signaling, TRIM21 reduces the production of type I IFNs and enhances HSV-3 replication in corneal epithelial cells (Tan and Xia, 2020) (**Figure 2**).

In HSV-1 infection, *Trim14^−/−^* mice were more susceptible to viral replication than wild-type mice (Chen et al., 2016b). Further studies revealed that TRIM14 is induced by type I IFNs. The TRIM14 inhibits cGAS ubiquitination by recruiting USP14 and reduces HSV-1 replication. Additionally, TRIM14 stabilizes cGAS, inhibits the p62-mediated cGAS autophagic degradation, and promotes the type I IFN signaling by recruiting USP14 to cleave the K414-linked ubiquitination at K48 of cGAS (Chen et al., 2016b) (**Figure 2**).

The relationship between cGAS-STING and autophagy is a fascinating area of research. The cGAS-STING signaling pathway can activate the autophagy-lysosome pathway. Conversely, autophagic lysosomes can degrade proteins ubiquitinated by the E3 ubiquitin ligase family. This process inhibits IFNs and promotes viral replication. In the development of antiviral drugs, we can consider designing drugs that target LC3 lipidation, as well as drugs that target molecules associated with the cGAS-STING signaling pathway (such as cGAS, TBK1 and IRF3) to reduce viral replication, thereby improving the efficacy of drug therapy for viral diseases.

### 3.2 Influence of viral replication through the autophagic PI3K/AKT/mTOR pathway

#### 3.2.1 Activation of autophagy enhances viral replication via the PI3K/AKT/mTOR pathway

The PI3K/AKT/mTOR pathway is an important intracellular signaling pathway involved in various cell life cycle activities, including autophagy (Aoki and Fujishita, 2017). Beclin 1 is a component of the class III phosphatidylinositol 3-kinase (PI3K-III) complex that regulates the production of phosphatidylinositol 3-phosphate (PI(3)P) (McKnight and Zhenyu, 2013). It is also involved in the recruitment of proteins necessary for autophagosome formation, and participates in the phagosome elongation process. Rubicon is a component of PI3KC3, and the interaction between rubicon and PI3Ks triggers the initiation of LAP. Moreover, the rubicon interacts with cGAS to inhibit DNA stimulation, thereby inhibiting cGAMP synthesis and type I IFN production (Funderburk et al., 2010; Kang et al., 2011; Stolz et al., 2014). Studies have shown that various viruses, such as HPV, HSV, and cytomegalovirus (CMV), can manipulate autophagy signaling pathways (Waisner and Kalamvoki, 2019). These pathways involve the Atg family, the PI3K signaling pathway, and membrane sources. Ultimately, this manipulation modulation of the delicate balance between autophagy and cell survival (Paul and Munz, 2016; Tsuboyama et al., 2016).

Sorting nexin 5 (SNX5) contains a Bin/amphiphysin/Rvs (BAR) domain. Interestingly, HSV-1 infection and replication were also increased in *Snx5^-/-^* MEFs compared with to wild-type cells. SNX5 has been shown to utilize the BAR structural domain to induce membrane bending when HSV-1 enters the endosomes (Gallop and McMahon, 2005; Mim and Unger, 2012; Dong et al., 2021). SNX5 interacts with beclin 1 and the Atg14-containing class III phosphatidylinositol-3-kinase (PI3KC3) complex 1 (PI3KC3-C1) to increase the lipid kinase activity of purified PI3KC3-C1. These findings explain an environment- and organelle-specific mechanism for initiating autophagy during viral infection (Dong et al., 2021).

During the early stages, human papillomavirus type 16 (HPV16) replication promotes protein synthesis and inhibits autophagy to facilitate viral infection via the interaction of the HPV16 PsV with epidermal growth factor receptor (EGFR) and epidermal growth factor receptor (KGFR) in HaCaT cells (human keratinocytes), which leads to the rapid activation of the PI3K/Akt/mTOR pathway and the impairment of autophagy (Surviladze et al., 2013).

The hepatitis B virus (HBV) X protein (HBx) inhibits autophagy, and its induction significantly increases DNA damage and nuclear translocation of ENDOG (Chao et al., 2021). Some studies have found that HBx induces endoplasmic reticulum stress and autophagy in host cells, promoting HBV replication and secretion through the AP-LE/MVB axis (Chao et al., 2021; Wang et al., 2022). Glucosamine (GlcN) promotes HBV replication through a dual mechanism that inhibits autophagic degradation and the blocks of mTORC1 signal transduction. Specifically, GlcN hinders lysosomal acidification through its amino group, thereby blocking autophagosome degradation. In addition, GlcN exhibits feedback inhibition of the mTORC1 signal in a GTPase-dependent manner involving the Ras-associated GTP-binding protein A (RAGA), leading to a further increase in autophagosome formation (Lin et al., 2020).

#### 3.2.2 Autophagy counteracts viral replication through the PI3K/AKT/mTOR pathway

On one hand, autophagic activity is regulated by the level of p62. On the other hand, p62 can also negatively regulate cellular autophagic activity by activating the mTORC1 signaling pathway. KSHV activates mTOR and its targets 4EBP1 and ULK1 and reduces bulk macroautophagy and mitophagy (Santarelli et al., 2020) (**Figure 2**).

Phagocyte nucleation is impelled by the induction of the beclin 1-PI3KIII complex. It has been revealed that porcine circovirus type 2 (PCV2) infection hampers the induction of IFN-*β*, both *in vitro* and *in vivo* (Li et al., 2021). In PCV2-infected cells, cGAS phosphorylation at S278 promotes K389-linked polyubiquitination of cGAS at K48, which is the recognition signal for the ubiquitin-binding structural domain of histone deacetylase 6 (HDAC6). This leads to the autophagic degradation of PCV2-infected cells and the degradation of cellular cGAS (Wang et al., 2021a). In the case of PCV2-infected PK-15 cells, the AMPK and extracellular signal-related kinase (ERK) 1/2 negatively regulates the mTOR pathway by phosphorylating TSC2, leading to autophagy induction through the AMPK/ERK/TSC2/mTOR signaling pathway (Zhu et al., 2012).

During the early stages of infectious spleen and kidney necrosis virus (ISKNV)-infected CPB cells, the PI3K, AKT, p53, and mTOR were significantly upregulated in cells. The activation of the PI3K/AKT/p53 factor to significantly reduce host autophagy while increasing the expression of immune-related genes in CPB cells, ultimately inhibiting the ISKNV replication (Li et al., 2017; Zhang et al., 2022a) (**Figure 1**).

Additionally, the neuroviral protein ICP34.5 encoded by HSV-1 binds to beclin 1 and inhibits the mammalian autophagy protein beclin 1. A mutant HSV-1 virus lacking the beclin 1-binding domain of ICP34.5 fails to inhibit autophagy in neurons and demonstrates impaired ability to cause lethal encephalitis in mice (Orvedahl et al., 2007; Qian et al., 2017) (**Figure 1**).

Activation of the PI3K/AKT/mTOR pathway promotes cells proliferation and protein synthesis, while inhibition of this pathway triggers autophagy. Several studies have shown that viral infection can increase viral replication by activating the PI3K/AKT/mTOR pathway, thereby accessing additional cellular resources. In summary, investigating the effect of autophagy induction via the PI3K/AKT/mTOR pathway on viral replication can improve our comprehension of viral infection mechanisms and provide a foundation for the creation of new antiviral approaches.

### 3.3 Autophagy inhibits viral replication through the NF-*κ*B signaling pathway

OPTN is highly homologous to the NF-*κ*B essential modulator (NEMO), and linear ubiquitination of the OPTN UBD inhibits NF-*κ*B (Zhu et al., 2007). The OPTN binds to interleukin 1 (IL-1) receptor-associated kinase 1 (IRAK-1), and OPTN effectively blocks TRAF6 polyubiquitination in response to IL-1*β* and toll-like receptor stimulation, thus further inhibiting NF-*κ*B activation. Furthermore, OPTN regulates protein and membrane cargo transport, and helps deliver ubiquitin cargo to autolysosome (Richter et al., 2016). Specifically, the *OPTN* gene regulates the transport of proteins and membrane cargo from the trans-Golgi compartment to the plasma membrane (Slowicka et al., 2016; Outlioua et al., 2018; Toth and Atkin, 2018). Phosphorylation of OPTN by TBK1 enhances its binding to Ub chains and promotes selective autophagy of damaged mitochondria (Richter et al., 2016).

Interestingly, in murine cytomegalovirus, the M45 protein interacts with NEMO to target autophagosomes and it is ultimately degraded in lysosomes, blocking Toll-like receptor (TLR) and IL-1 receptor-dependent NF-*κ*B activation at the level of the IKK complex, and inhibiting the inflammatory cascade (Fliss et al., 2012). Moreover, the KSHV-encoded FLICE inhibitory protein (vFLIP) K13 binds to the IKK complex, finally leading to the activation of the NF-*κ*B pathway. Moreover, vCFLAR/vFLIP has been shown to be a potent autophagy inhibitor by binding to and inhibiting Atg3, which prevents LC3 targeting to phagophores (Leidal et al., 2012).

During the early stages, the HSV-1-infected cell protein 0 (ICP0) inhibits SQSTM1/p62 and OPTN. It has been revealed that the cells lacking either p62 or OPTN are able to exhibit a greater antiviral response, whereas the cells expressing exogenous p62 show reduced virus yields (Waisner and Kalamvoki, 2019). The above data demonstrate that the suppression of SQSTM1/p62 and OPTN is a viral strategy to counteract the host defenses.

### 3.4 Autophagy affects viral replication through the eIF2α kinase signaling pathway

eIF2α kinase, a regulator of stress-induced translational control processes, is also involved in regulating stress-induced autophagy. It has been demonstrated that divergent stress stimuli stimulate the eIF2α kinase-dependent translational arrest and the eIF2α kinase-dependent autophagy (Talloczy et al., 2002). The elF2α kinase pathway regulates the starvation- or virus-induced autophagy (B'Chir et al., 2013). During HSV-1 infection, the activation of the IFN response and eukaryotic translation initiation factor 2α kinase 2 (eIF2AK2) leads to the phosphorylation of eIF2α and the induction of autophagy (Talloczy et al., 2002; Bullido et al., 2008). Additionally, during the early stages of viral infection, USP14 directly binds to the ubiquitin chain on VP16 through its UBL domain. Inactivation of USP14 stimulates the eIF2AK3/PERK and ERN1/IRE1-mediated signaling pathways, resulting in VP16 degradation via the SQSTM1/p62-mediated autophagy, thus inhibiting herpesvirus proliferation (Ming et al., 2022). The HSV-1 ICP34.5 protein binds to beclin 1 to form a complex inhibiting autophagy (Orvedahl et al., 2007; Leib et al., 2009). The HSV-1 mutant lacking ICP34.5 triggers autophagy by activating the eIF2AK2/protein kinase RNA (PKR) activation pathway to evade, resist antiviral mechanisms and enhance viral replication (Orvedahl et al., 2007). IFN-induced PKR enhances autophagy in response to HSV-1 infection, and HSV-1 neurovirulence protein ICP34.5 inhibits this response. The HSV-1 neurovirulence protein ICP34.5 inhibits autophagy regulated by PKR or elF2α (Talloczy et al., 2002) (**Table 1**).

**Table 1:** Viral proteins and the autophagy pathway.

Viruses	Viral proteins	Atg proteins	Effects
ASFV	MGF505-7R	ULK1	MGF-505-7R promotes the expression of ULK1 to degrade STING.
SARS-CoV-2	ORF3a	Rab7	ORF3a-VPS39 blocks the fusion of autophagosomes and lysosomes, eventually inhibiting autophagy.
	ORF7a	SNAP29	ORF7a cleaves the SNAP29 protein by activating caspase 3, and hampers the fusion of autophagosomes with lysosomes.
	ORF10	NIX	ORF10 induces mitophagy-mediated MAVS degradation by binding to NIX.
PCV2	Cap	mTOR	The cap protein leads to autophagy by activates the PI3K/AKT signaling and PKC*δ* signaling.
HSV	ICP34.5	Beclin 1	ICP34.5 binds to beclin 1, thereby inhibiting autophagy.
HSV-1	ICP0	SQSTM1/p62	HSV-1 ICP0 protein inhibits SQSTM1/p62 and OPTN.
ZIKV	NS4A/NS4B	Akt	NS4A and NS4B block Akt activation and potentially modulating Akt post-translational modifications.
KSHV	vFLIP	Atg3	vFLIP binds to and inhibiting Atg3, thereby preventing LC3 targeting to phagophores.
	XPO1	SQSTM1/p62	XPO1 promotes the activation of TBK1 and IRF3 through inhibition of nuclear aggregation of SQSTM1/p62.
SVA	2AB	LC3	2AB inhibits the autophagy process by degrading MARCH8 and LC3.
EBV	BPLF1	SQSTM1/p62	BPLF1 binds to SQSTM1/p62, reducing its ubiquitination and inhibiting autophagy.
VSV	/	Atg5-Atg12	Atg5-Atg12 conjugate interacts with IPS-1 and RIG-I to inhibit type I IFN production.
HPIV3	Phosphoprotein (P)	SNAP29	HPIV3 P protein binds to SNAP29, blocking its interaction with protein 17 and stopping host SNARE protein-mediated autophagosome-lysosome fusion.
IAV	M2	Beclin 1	IAV M2 protein colocalizes with autophagosomes and plays an important role in inhibiting autophagosomal fusion.

However, a recent study indicates that the IFN-*β*-inducible SCOTIN (an ER-resident protein, also known as SHISA5) recruits the HCV NS5A for autophagic degradation, thereby suppressing HCV replication (Kim et al., 2016). However, the human cytomegalovirus (HCMV)-encoded protein IRS1 inhibits autophagy by interacting with beclin 1, independently of the eIF2SI kinase eIF2/PKR (Mouna et al., 2016) (**Figure 2**).

### 3.5 Other pathways

Secretory autophagy is nonlytic autophagy. In this process, autophagic cargo is secreted from autophagic vesicles into the extracellular compartment (Ponpuak et al., 2015). Previous studies have shown that secretory autophagy is associated with both immune response and inflammation (Deretic et al., 2013). GAL9, an IFN-induced innate antiviral factor, can promote autoubiquitination of RNF13, which leads to the recruitment of p62 and LC3 to initiate autophagy. GAL9 works in tandem with viperin that degrades the HBV core protein HBC, and enhances the GAL9-induced antiviral activity, which may offer therapeutic interventions against HBV (Miyakawa et al., 2022) (**Figure 2**). The virus-infected cells release large numbers of vesicles derived from autophagosome (Feng et al., 2013; Chen et al., 2015). Currently, there are fewer studies on secretory autophagy. Researchers can focus their studies on revealing the molecular mechanisms by which viruses utilize the secretory autophagy to promote the assembly and release of viral particles.

The SQSTM1/p62 protein has a ubiquitin-binding domain and a LC3 interaction motif, the ubiquitination of SQSTM1/p62 modifies autophagic activity (Pankiv et al., 2007). The ubiquitin uncoupling enzyme in the N-terminal domain of the large capsid protein of Epstein-Barr virus (EBV), HCMV, and KSHV, regulates selective autophagy by inhibiting the activity of the autophagy receptor SQSTM1/p62 (Yao et al., 2023) (**Figure 1**). EBV BPLF1 is a deubiquitinating enzyme that can directly bind to the autophagy receptor SQSTM1/p62, reduce the ubiquitination level of SQSTM1/p62, and finally inhibit selective autophagy (Yla-Anttila et al., 2021). In the replication cycle of EBV, autophagy is activated in the early stage of EBV reactivation and inhibited in the subsequent stage (Granato et al., 2014). Inhibition of autophagy can significantly up-regulate the expression and replication of EBV genes and increase the production of EBV particles (Hung et al., 2014; Nowag et al., 2014). Additionally, autophagosome-lysosome fusion is reduced in a Rab7-dependent manner, leading to autophagosome accumulation and inhibiting autophagy (Granato et al., 2014; De Leo et al., 2015) (**Table 1**).

Numerous studies have shown that viral proteins can influence autophagy and the innate immune response during DNA virus infection through multiple pathways. The interactions between viral infection and the host antiviral mechanisms ultimately result in either enhanced or attenuated viral replication. In the future, we will investigate how innate immunity interacts with autophagy. This work will improve our understanding of the molecular mechanisms underlying host-generated antiviral resistance during viral infections and enable more precise targeting of antiviral drugs.

## 4 Autophagy regulates innate immunity and participates in the replication of RNA viruses

Cytoplasmic DNA can interact with the DNA sensor cGAS. The DNA induces IFN-*β* upon cGAMP production and activation of STING and TBK1/IKK*ϵ* (Yoneyama et al., 2005; Ablasser and Hur, 2020). In addition, RNA can bind directly to the RNA sensors retinoic acid-inducible gene-I (RIG-I), MDA5 or may promote increased mitochondrial permeability and release of oxidized mitochondrial DNA into the cytoplasm. RIG-I binds preferentially to 5′-triphosphorylated viral RNA with short dsRNA stretches (Hornung et al., 2006; Kato et al., 2006; Kato et al., 2008). RIG-I is responsible for recognizing influenza virus, Sendai virus (SeV), vesicular stomatitis virus (VSV), and Newcastle disease virus (NDV) (Kato et al., 2006; Kato et al., 2008). Whereas, MDA5 exhibits a higher affinity for longer dsRNA and primarily recognizes picornaviruses, such as encephalomyocarditis virus (EMCV). Several RNA viruses can be sensed by both RIG-I and MDA5 (Lian et al., 2018). Upon binding to viral RNA, both RIG-I and MDA5 are recruited to the mitochondria-localized articulatory protein mitochondrial antiviral-signaling protein (MAVS). The RNA-sensing pathway induces IFN-*β* upon activation of mitochondria-associated MAVS and downstream activation of signaling molecules such as TRAF3, TRAF6, TBK1/IKK*ϵ*, and NF-*κ*B (Yoneyama et al., 2015; Crow et al., 2019; Rehwinkel and Gack, 2020).

Both DNA and RNA viruses can employ unique strategies to modulate autophagic responses, allowing the viruses to evade damage or replicate by utilizing the structure and nutrients provided by autophagy. A comprehensive understanding of the complex relationship between RNA virus infections and the host innate immunity is critical to effective control viral infections.

### 4.1 Autophagy and IFN production in response to RNA viral infections

SARS-CoV-2, the virus responsible for COVID-19, has been shown to promote autophagy and suppressing the type I IFN response (Hui et al., 2021). It has been shown that ubiquitination of the SARS-CoV-2 ORF7a protein to inhibit the IFN response. Additionally, the ORF7a protein triggers autophagy through the AKT-mTOR-ULK1-mediated pathway (Kang et al., 2011). Overexpression of the SARS-CoV-2 ORF10 protein significantly reduces the expression of type I IFNs (Cao et al., 2021). At the same time, ORF10 is translocated to mitochondria by interacting with the mitophagy receptor Nip3-like protein X (NIX), and induces mitophagy via its interaction with both NIX and LC3B (Li et al., 2022). ORF10-induced autophagy can degrade the MAVS and promote SARS-CoV-2 replication. In short, SARS-CoV-2 ORF10 induces degradation of MAVS by binding to NIX (Li et al., 2022) (**Table 1**). The above evidence suggests that in order to favor its own replication, the viral protein encoded by SARS-CoV-2 induces autophagy and reduces type I IFN production.

The 2AB protein of Senecavirus A (SVA) inhibits MAVS in a membrane associated ring-CH-type finger 8 (MARCHF8)-dependent manner. Concurrently, the 2AB, MARCHF8, and LC3 proteins form a complex. The 2AB inhibits the autophagy process by degrading MARCH8 and LC3. In addition, MARCHF8 acts as a positive regulator of the type I IFN signaling pathway, and degradation of MARCHF8 can effectively inhibit type I IFN activation and promote SVA replication (Sun et al., 2022) (**Table 1** and **Figure 1**). Similar to SARS-CoV-2, SVA is able to utilize autophagy and inhibit type I IFN responses to evade the host immune system.

Moreover, the NOD-like receptor family member X1 (NLRX1) can suppress the RIG-I-mediated IFN-*β* expression and promote VSV-induced autophagy (Jordan and Randall, 2012; Lei et al., 2012; Chiramel et al., 2013; Paul and Munz, 2016). The viral RNA of VSV can be sensed by the cytoplasmic RIG-I-like receptors (RLRs) to produce type I IFNs by activating the IFN-*β* promoter stimulator 1 (IPS-1). However, when VSV infects host cells, the Atg5-Atg12 conjugate interacts with IPS-1 and RIG-I, inhibiting the CARD-mediated signaling and leading to the inhibition of type I IFN production, thus blocking the innate antiviral immune responses (Jounai et al., 2007; Tal et al., 2009) (**Table 1** and **Figure 2**). The discovery highlights the crucial involvement of NLRX1 in controlling viral infections, which modulates the host immune response by inhibiting IFN-*β* expression and promoting autophagy.

During the early stages of dengue virus (DENV) replication, autophagy is induced to mobilize the necessary membrane and lipids (Welsch et al., 2009; Heaton and Randall, 2010). DENV has evolved to inhibit STING activity, where NS2B3 protease cleaves STING to inhibit IFN induction. Meanwhile, NS2B degrades cGAS, and autophagy induced by STING activation supports mutant DENV replication in infected cells (Aguirre et al., 2012; Aguirre et al., 2017; Ng et al., 2022). The Zika virus (ZIKV) NS4A and NS4B proteins deregulate the Akt-mTOR signaling in human fetal neural stem cells to inhibit neurogenesis and induce autophagy (Liang et al., 2016) (**Table 1** and **Figure 1**).

These findings reveal strategies by which viruses utilize mechanisms of autophagy and suppression of immune response to evade host immune attack and promote viral replication. Studying these mechanisms will help us better understand the immune evasion strategies of viral infections and provide new ideas for developing therapeutic approaches against viral infections.

### 4.2 Reducing autophagosome-lysosomal fusion can increase viral production

It has been found that some viruses evade autophagy by reducing the fusion of autophagosomes and lysosomes in host cells. The SARS-CoV-2 ORF3a interacts with VPS39 and is colocalized in the lysosomes. The ORF3a-VPS39 inhibits the binding of the homotypic fusion and protein sorting (HOPS) to Rab7, hinders the assembly of fusion machinery, and leads to the reduction of autophagosomes. The ORF3a strongly inhibits autophagic flux by blocking the fusion of autophagosomes and lysosomes (Zhang et al., 2021). The SARS-CoV-2 ORF7a cleaves the SNAP29 protein by activating caspase 3. The degradation of the SNAP29 protein hampers the fusion of autophagosomes with lysosomes, consequently promoting viral replication (Hou et al., 2023). The phosphoprotein of human parainfluenza virus type 3 (HPIV3) can inhibit autophagy to increase virus production. The virus phosphoprotein (P) binds to SNAP29 and inhibits its interaction with the synaptic fusion protein 17, thereby preventing the host SNARE protein-mediated autophagosome-lysosome fusion (Ding et al., 2014) (**Table 1** and **Figure 1**).

### 4.3 Other types of autophagy reactions

Recently, the significance of non-canonical autophagy has been highlighted in the host antiviral immune responses. For example, LC3C contributes to the Vpu-mediated evasion of BST2/tetherin restriction on HIV-1 release through a non-canonical autophagy pathway (Madjo et al., 2016). Additionally, mice with systemic loss of non-canonical autophagy exhibited heightened susceptibility to pathogenic Influenza A virus (IAV), and non-canonical autophagy hinders the fusion of IAV with endosomes and dampens activation of the IFN signaling pathway (Wang et al., 2021). The IAV matrix protein 2 (M2), which is colocalized with autophagosomes, is able to prevent the fusion of autophagosomes with lysosomes by interacting with beclin 1 (Gannage et al., 2009; Zhong et al., 2009; Wang et al., 2019). The M2 protein interacts with MAVS and enhances the MAVS-mediated innate immunity. Moreover, the M2-induced production of reactive oxygen species (ROS) plays a critical role in activating autophagy and the MAVS signaling pathway (Wang et al., 2019). IAV infection also induces the formation of autophagosomes to facilitate viral replication (Zhou et al., 2009).

In summary, some viruses hinder the maturation of autophagosomes to prevent degradation. They subsequently exploit the autophagosome membrane to facilitate the packaging and/or release of viral particles.

Secretory autophagy, a process in which the autophagosome carrier is secreted into the extracellular environment instead of being degraded, has emerged as a prominent research area in recent years. It has been reported that several small RNA viruses can evade cellular immune response with extracellular vesicles (EVs) containing the autophagy regulatory protein LC3 (Sin et al., 2017; van der Grein et al., 2019). In the case of EMCV, its leader protein induces secretory autophagy during infection, promoting the release of viral particles in EVs (van der Grein et al., 2022).

Over the years, researchers have extensively studied the link between autophagy, type I IFN response and RNA virus infection. They have found that some RNA viruses can evade the host immune defense by interfering with autophagy and type I IFN response, leading to viral replication and transmission. Consequently, a comprehensive investigation of the regulatory mechanisms of autophagy and type I IFN responses in RNA virus infections can help to uncover the interactions between virus and host. Researchers also hope to discover new drugs or therapeutic strategies to inhibit viral replication and propagation by activating autophagy and type I IFN response, thus effectively controlling RNA virus infection.

## 5 Conclusions and prospects

Autophagy, an evolutionarily conserved degradative process, is used to maintain organismal homeostasis and promote the clearance of intracellular waste and invading pathogens by the host immune system, including disrupting viral structure, modulating the inflammatory response, influencing IFN production, and degrading viral components or particles. Some viruses employ unique strategies to regulate the autophagy response, allowing viruses to evade destruction or utilize the structure and nutrients provided by autophagy for their replication. On one hand, host cells can effectively counteract viral replication by regulating the signaling molecules of the IFNs and autophagy pathways through multiple pathways. On the other hand, viruses inhibit innate immune responses by regulating the expression of signaling molecules associated with the autophagy pathway, thereby accelerating autophagic degradation of cellular components. A comprehensive understanding of the mechanisms by which autophagy regulates viral replication and the interactions between autophagy and the IFN signaling pathway are needed.

This review reveals the complex relationship between viral infections and host autophagy, which is an attractive research field. Cross-regulation of autophagy and IFNs triggered by viral infection is a promising area. Further work is needed to fully understand the involvement of autophagy and IFN signaling pathways in the pathogenesis of resistance to viral infections. Importantly, current studies are mostly focused on typical autophagy, with less research on viral infection and non-canonical autophagy. Most of the existing studies evaluate the antiviral effects of autophagy *in vitro*, and it’s necessary to validate the effects of autophagy on viral replication *in vivo*, which is more important for the development of autophagy-regulated antiviral therapeutic strategies and vaccines.

## Author contributions

HZ: Writing – original draft. TW: Writing – review & editing. DL: Writing – review & editing. LP: Writing – review & editing. YS: Writing – review & editing. H-JQ: Writing – review & editing.
